# Case report: A Chinese boy with facial dysmorphism, immunodeficiency, livedo, and short stature syndrome

**DOI:** 10.3389/fped.2022.933108

**Published:** 2022-08-22

**Authors:** Lihong Jiang, Xin Chen, Jiaqi Zheng, Meilin Wang, Hui Bo, Geli Liu

**Affiliations:** ^1^Department of Pediatrics, Tianjin Medical University General Hospital, Tianjin, China; ^2^Jinghai Clinical College of Tianjin Medical University, Tianjin, China; ^3^Department of Pediatrics, Jinghai District Hospital, Tianjin, China

**Keywords:** FILS syndrome, *POLE*, short stature, dysmorphism, immunodeficiency

## Abstract

Facial dysmorphism, immunodeficiency, livedo, and short stature (FILS) syndrome is a rare autosomal recessive disease. In this study we reported the first Chinese patient with FILS syndrome. The patient had short stature and suffered from recurrent respiratory infections up to the age of 4 years. Other symptoms of the disease included livedo on the inner side of upper limbs and thigh skin, prominent forehead, low anterior and posterior hairline, short and down-slanting palpebral fissure, low-set ears, long nasal tip and columella, and a small mouth with irregular teeth. A whole exome sequencing (WES) was performed and revealed two variants within the polymerase ε (*POLE*) gene. One of the variants was a splicing variant (c.5811 + 2T > C) derived from the mother, while the other was a nonsense variant (c.2006G > A) derived from the father. These two variants were not reported in previous FILS syndrome cases. Therefore this case provides further insight into the *POLE* gene variant spectrum that enriches the clinical phenotype.

## Introduction

DNA polymerases are a group of enzymes that catalyze the synthesis of DNA during replication. There are 16 different types of DNA polymerases in the human body. However, the efficient and accurate replication of the eukaryotic nuclear genome depends heavily on the DNA polymerases (Pol) α, δ, and ε. The δ and ε DNA polymerases are responsible for most of the DNA replication tasks. They are also responsible for the activity of the 3’-5’ exonuclease, which is involved in reducing errors during DNA replication. Although the DNA-pol δ and ε have similar functions, they are distributed differently (1, 2). The gene family coding DNA-pol ε is known as polymerase ε (*POLE*) and includes *POLE1*, *POLE2*, *POLE3* and *POLE4*, which encode four subunits of the DNA-pol ε. The facial dysmorphism, immunodeficiency, livedo, and short stature (FILS) syndrome is an extremely rare autosomal recessive disease caused by mutations in the *POLE* gene. FILS syndrome was first reported in a French consanguineous family in 2012. In this study, we reported the first Chinese patient with FILS syndrome and the impact of the *POLE* gene variant on the clinical phenotype.

## Case report

**FIGURE 1:**
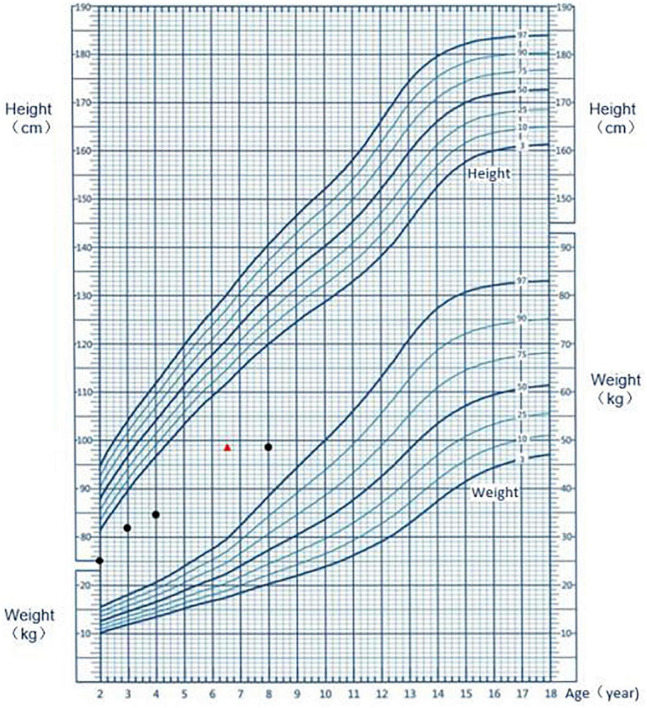
Growth chart of the patient. The black dot represents the height at the respective age; the red triangle indicates the height at the corresponding bone age.

An 8-year-old Chinese boy was admitted to hospital to due to delayed growth. He was born full-term, with a weight of 2.45 kg and a height of 48 cm. At the gestational age of 8 months, a fetal B-ultrasound showed that his limbs were short. His mother was diagnosed with hypothyroidism during pregnancy. The mother was treated with levothyroxine sodium tablets and the thyroid function was normal during the rest of the gestational period. His height was 67 cm (–3.52SDS) at 1 year of age, 75 cm (-3.97SDS) at 2 years old, 82 cm (-3.89SDS) at 3 years old, and 84.5 cm (-5.02SDS) at 4 years old (Figure 1). The patient could speak at the age of 14 months and walk at the age of 16 months. He was behind by about 1 month in the development milestones compared to normal children. The patient attended grade two in primary school and had a good academic performance. The patient’s vaccination status was appropriate. He suffered from recurrent respiratory infections up to the age of 4 years. His parents denied consanguinity. The child also has an elder brother. The fetal B-ultrasound suggested that his elder brother had intrauterine growth retardation and short limbs. The patient’s brother was born at full term, with a birth weight of 2 kg and a birth length of 46 cm. However, he died of possible pneumonia 50 days after birth.

**FIGURE 2:**
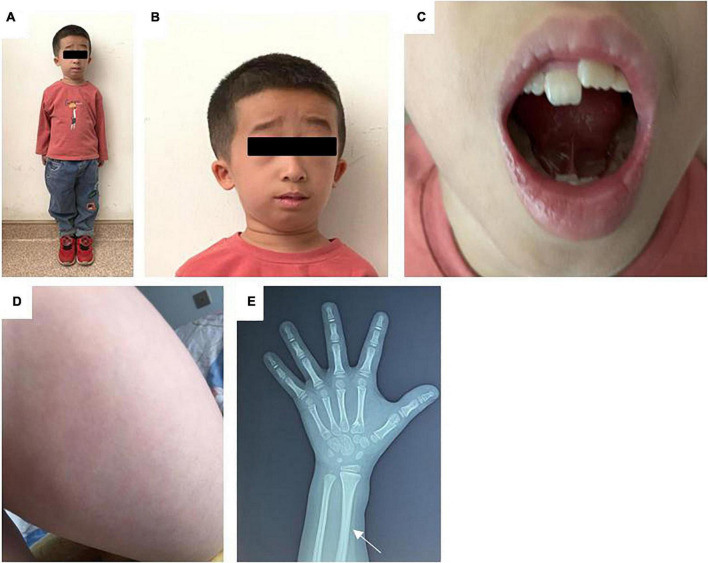
**(A)** Full body photograph of the patient. **(B)** Photograph of the child’s face: zygomatic arch dysplasia, a high forehead, low anterior hairline, low-set ears, a long nasal tip and columella, and a small mouth. **(C)** Photograph of the child’s irregular teeth. **(D)** Photograph illustrating the livedo on the skin of the patient’s thigh. **(E)** X-ray of the left hand. The white arrow indicate a thickened radial cortex, a narrow medullary cavity, and a bent radial diaphysis.

On admission to the hospital, the child had a height of 86 cm (-5.81SDS), weighted 15 kg (< P_3r*d*_), and had a body mass index (BMI) of 15.6 kg/m^2^(P_25–50t*h*_). His head circumference, finger distance, and sitting height were 51, 90, and 59 cm, respectively. The skin on the inner side of the upper limb and thigh had signs of livedo. He had various facial deformities typical of FILS syndrome, including zygomatic arch dysplasia, a high forehead, low anterior and posterior hairline, short and down-slanting palpebral fissure, low-set ears, a long nasal tip and columella, and a small mouth. The patient also had irregular teeth. His limbs were short (Figure 2). He had a 2 cm long webbed penis and a testicular with a volume of 0.5 mL.

Routine blood tests showed that the electrolytes, liver function, renal function, thyroid function, adrenocortical function, and fasting glucose and insulin levels were normal. Chromosome karyotype analysis revealed a 46 XY. The insulin-like growth factor 1 (IGF-1), insulin-like growth factor binding protein 3 (IGFBP-3), and 25-hydroxyvitamin D levels in the blood were normal. All immunoglobulins (Ig) except IgG4 were normal. The alpha-fetoprotein (AFP), human chorionic gonadotropin (HCG), and carcinoembryonic antigen (CEA) levels were normal. Conversely, the carbohydrate antigen 199 (CA 199) level was elevated (Table 1). The growth hormone stimulation test showed a peak level of 13.4 ng/mL, indicating no growth hormone deficiency. The lymphocyte subset analysis showed that the count and proportion of the T cells, B cells, and natural killer (NK) cells were normal. A testicular B-ultrasound showed that the volumes of the left and right testicles were 0.40 and 0.47 mL, respectively. An X-ray of the left hand revealed a thicker cortex with a narrowed medullary cavity and a bent radial diaphysis (Figure 2). Bone age was delayed by 1.5 years. Pituitary magnetic resonance imaging (MRI) revealed a small flat pituitary with a height of about 2.5 mm. No abnormal MRI signals were detected from the pituitary.

**TABLE 1:** Summary of laboratory findings.

	Laboratory findings	Reference range
IGF-1 (ng/mL)	182.00	40.00–255.00
IGFBP-3 (μg/mL)	7.42	0.70–7.70
25OHD (nmol/L)	52.26	15.5–113.75
IgG (mg/dL)	678.00	300.00–1300.00
IgG1 (g/L)	5.32	4.05–10.11
IgG2 (g/L)	1.96	1.69–7.86
IgG3 (g/L)	0.17	0.11–0.85
IgG4 (g/L)	**0.01**	0.03–2.01
IgA (mg/dL)	74.10	29.00–270.00
IgE (IU/mL)	86.00	<87.00
IgM (mg/dL)	63.60	50.00–260.00
AFP (ng/mL)	0.78	0.00–8.78
CEA (ng/mL)	1.64	0.00–5.00
CA199 (U/mL)	**67.13**	0.00–37.00
HCG (mIU/mL)	<1.20	0.00–5.00

IGF-1, insulin-like growth factor 1; IGFBP-3, insulin-like growth factor-binding protein 3; 25OHD, 25 hydroxyvitamin D; AFP, Alpha-fetoprotein; CEA, carcinoembryonic antigen; CA 199, carbohydrate anti 9; HCG, human chorionic gonadotropin. Ig, immunoglobulin the values highlighted in bold are not within the normal range.

**FIGURE 3:**
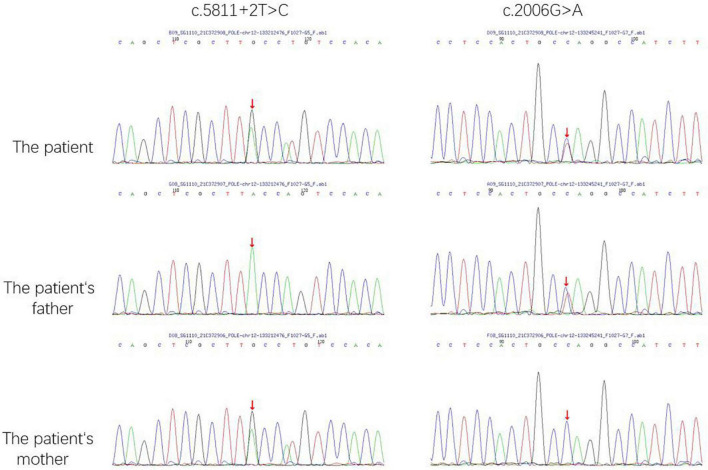
The *POLE* gene sequencing map of the patient and his parents. The red arrow indicate the sites of mutations.

The whole exome sequencing (WES) revealed compound heterozygous pathogenic variants in the proband’s *POLE* gene. One variant was a splicing variant (c.5811 + 2T > C). The other variant was defined as a nonsense variant (c.2006G > A), resulting in a truncated protein (p.W669X). According to the American College of Medical Genetics and Genomics (ACMG) guidelines, these variants were classified as likely pathogenic (PVS1 + PM2-p) (Figure 3).

## Discussion

FILS syndrome (OMIM 615139) is a rare autosomal recessive disease caused by mutations in the *POLE* gene. A literature search on PubMed up to March 2022 using the keywords “FILS syndrome” and “POLE” revealed only 3 studies on FILS syndrome with a total of 16 patients. In this study, we reported the first Chinese patient with FILS syndrome.

The physical appearance, growth patterns, and immune deficiency of patients with FILS syndrome can vary widely. In our study, in addition to having the characteristic facial dysmorphism, the patient also had irregular teeth, possibly caused by skeletal dysplasia. Other signs included abnormalities in the reproductive organs such as micropenis and micro-testis. Pachlopnik Schmid et al. (3) reported on 14 patients from a closely related family with FILS syndrome. Although these patients were born with normal weight and length, growth delays were observed in early childhood, resulting in short stature in adulthood (3). In the case report of Thiffault et al. the Palestinian patient was diagnosed with intrauterine growth retardation (4). Similarly, in our case, the B-ultrasound performed at the gestational age of 8 months showed that the fetus had short limbs and low birth weight (2.45 kg) and height (48 cm) indicative of intrauterine growth retardation. The monitoring of the patient’s height after birth showed that growth delay increased with age (Figure 1). Patients with FILS syndrome also tend to have recurrent upper respiratory tract infections, pneumonia, sinusitis, and suppurative otitis media. Study also reported the patients had bronchiectasis caused by recurrent pneumonia infections and recurrent meningitis caused by streptococcus pneumonia (3). The increased susceptibility to infections is likely to be related to low IgG levels, particularly IgG2. IgG2 has an important role in triggering an immune response to polysaccharide antigen. Therefore patients with IgG2 deficiency are prone to recurrent respiratory infections such as *H. influenzae* infections and pneumonia by *S. pneumoniae*. The blood test of the Palestinian girl showed high IgA, normal total IgG and low IgM, IgG2, and IgG4, potentially leading to more serious infectious diseases (4). Conversely, the patient in our study had normal total IgG, IgG2, IgM, and IgA levels, except for IgG4, and therefore, the immunodeficiency symptoms in our patient were mild. The child had recurrent upper respiratory tract infections and pneumonia, which resolved after the age of 4 years. FILS syndrome is also associated with a wide range of skin phenotypes. Livedo on the face and bilateral upper and lower limbs after birth is the most common skin condition. Some other children present with other skin lesions, such as repeated itchy papules, skin thickening, and deep induration (5). In this case, the skin manifestation was typical but mild. Overall, we concluded that the clinical phenotype of this case was consistent with that reported in previous studies but with less immune deficiency and skin lesions, and severe short stature.

The Trios-WES identified compound heterozygous variants in the *POLE* gene. Pedigree genetic analysis revealed that the nonsense mutation (c.2006G > A) was inherited from his father while the splice site variant (c.5811 + 2T > C) was inherited from his mother. Both variants were novel and classified as likely pathogenic according to ACMG guidelines. This means that these two variants should be assume to disrupt gene function by leading to a complete absence of the gene product by lack of transcription or nonsense-mediated decay of an altered transcript. The c.5811 + 2T > C variant, resulted in errors during the splicing process and led to the 42nd exon’s removal. The c.2006G > A variant, which was observed in the DNA polymerase type-B family catalytic domain, affected the process of polymerase catalysis.

The main pathogenesis of FILS syndrome is the abnormal expression of DNA-pol ε, which is caused by variants in the *POLE* gene. The *POLE* gene, located in 12q24.33, encodes the catalytic subunit of DNA-Pol ε, which contains two domains: the polymerase domain and the exonuclease proofreading domain. The former mainly participates in the synthesis of leading chains during DNA replication, while the latter, together with DNA-pol δ and mismatch repair (MMR) protein, maintains the stability of the genome and plays a role in maintaining high-fidelity DNA replication and preventing mutations (1). Due to the different expression levels of POLE in different tissues, a decline in the polymerase activity of DNA-pol ε may restrict the proliferation of T cells, B cells, chondrocytes, osteoblasts, and skin fibroblasts (3), leading to pathological changes in immunity, bone and skin. Studies have shown that colorectal cancer and endometrial cancer are related to mutations in the DNA-pol ε exonuclease proofreading domain, and a considerable number of these patients also have the same *MMR* gene mutations (6, 7). Some studies found that the cell lines derived from patients with the *POLE* gene mutation had decreased cell proliferation and increased levels of DNA damage in cell culture (8). At present, it has been found that the phenotypes of FILS syndrome are mainly related to impaired polymerase activity of the DNA-pol ε and preferential expression of *POLE* in some tissues. However, no tumor susceptibility has been observed in any of the 16 patients with FILS syndrome reported so far. Although this patient did not have any gene mutations in the proofreading domain, the proofreading function of the DNA-pol ε might have been altered the stability and activity of the holoenzyme. As a result, these changes may still indirectly increase the risk of developing cancer.

Eleven out of the 14 members of the closely related family members with FILS syndrome in France had all the typical phenotypes of FILS syndrome, while the other three patients exhibited only two or three phenotypes. The genetic tests of the 14 patients in that study revealed homozygous nucleotide substitution in the 34th intron of the *POLE* gene (g.G4444 + 3A > G), resulting in the interruption of the G1-S phase of DNA replication (3). Thiffault et al. reported on a Palestinian female infant with the same variant of the *POLE* gene as patients from the consanguineous French family. She presented with more severe growth restriction and immunodeficiency, suggesting that the same variants in the *POLE* gene may resulted in different phenotypes. Eason et al. reported serious dermal lesions on a 6-year-old Hispanic boy with the homozygous variation of the *POLE* gene [C.100C > T (p.Arg34Cys)] (5). Two novel variants were noted in our case when compared with the previously reported cases. The irregular teeth were not reported in previous case reports on FILS syndrome. We also speculate that proband’s elder brother carried the same pathogenic gene but showed more serious intrauterine growth restriction (birth weight: 2 kg, length: 46 cm) and immune deficiency. However, their phenotypes differed, indicating that other mechanisms may be involved in this process, warranting further exploration.

Based on the findings of this case report and previous studies, we recommended monitoring the child for the development of specific diseases. At present, there is no definite conclusion on whether FILS syndrome caused by *POLE* will increase tumor susceptibility, but it is speculated that the risk of lymphoma and skin cancer will increase. Therefore, the child should be screened regularly for skin cancer and lymphoma. Bone lesions were found in 4 out of the 14 patients diagnosed with FILS syndrome in France. Radiographic examination of this child’s upper limbs revealed a thickened radial cortex, a narrow medullary cavity, and a bent radial diaphysis, which could have potentially been caused by inconsistent development rates between the ulna and radius. The increase in body weight during adulthood will also increase the skeletal load on lower limbs. Therefore regular radiographic examinations of the upper and lower limbs are recommended to exclude the presence of bone lesions. The CA199 tumor marker should be monitored to identify any possible cancers at early stage.

## Conclusion

FILS syndrome is a very rare disease. Although FILS syndrome has typical clinical characteristics, the phenotypic spectrum may vary widely. Further research is recommended to better elucidate the genetic mechanisms involved in the development of this disorder.

## Data availability statement

The original contributions presented in this study are included in the article/Supplementary material, further inquiries can be directed to the corresponding author.

## Ethics statement

The studies involving human participants were reviewed and approved by Authorization of the Ethical Committee of Tianjin Medical University General Hospital. Written informed consent to participate in this study was provided by the participants’ legal guardian/next of kin. Written informed consent was obtained from the individual(s) and minor(s)’ legal guardian/next of kin, for the publication of any potentially identifiable images or data included in this article.

## Author contributions

GL contributed to the conception and design of the study. LJ wrote the first draft of the manuscript. XC and JZ wrote sections of the manuscript. MW and HB collected clinical data. All authors contributed to manuscript revision, read, and approved the final version for submission.
