# Supplementary material for: Case report: A Chinese boy with facial dysmorphism, immunodeficiency, livedo, and short stature syndrome
Source: Front Pediatr. 2022 Aug 22;10:933108. doi: 10.3389/fped.2022.933108 (PMC9441657; doi:10.3389/fped.2022.933108)
Supplement: Supplementary file 1 [file Data_Sheet_1.docx]

GenBank accession numbers

BankIt2576693 Seq1 ON366697

BankIt2576693 Seq2 ON366698

BankIt2576693 Seq3 ON366699

BankIt2576693 Seq4 ON366700

BankIt2576693 Seq5 ON366701

BankIt2576693 Seq6 ON366702

BankIt2576693 Seq7 ON366703

BankIt2576693 Seq8 ON366704

BankIt2576693 Seq9 ON366705
